# FAM136A immunoreactivity is associated with nodal involvement and survival in lung adenocarcinoma in a Chinese case series.

**DOI:** 10.1080/21655979.2020.1735611

**Published:** 2020-03-01

**Authors:** Liufang Zhao, Chunlei Ge, Zhiwei Zhang, Hongyan Hu, Yi Zhang, Wentao Zhao, Ruilei Li, Baozhen Zeng, Xin Song, Gaofeng Li

**Affiliations:** a First Department of Head and Neck Surgery, The Third Affiliated Hospital of Kunming Medical University, Tumor Hospital of Yunnan Province, Kunming, Yunnan, 650118, P.R. China; b Department of Cancer Biotherapy Center, Tumor Hospital of Yunnan Province, Kunming, Yunnan, 650118, P.R. China; c Department of Pathology and Histotechnology, Tumor Hospital of Yunnan Province, Kunming, Yunnan, 650118, P.R. China; d Department of Gynecology, Tumor Hospital of Yunnan Province, Kunming, Yunnan, 650118, P.R. China; e Department of Medical Oncology, Tumor Hospital of Yunnan Province, Kunming, Yunnan, 650118, P.R. China; f Department of Thoracic Surgery, The Third Affiliated Hospital of Kunming Medical University, Kunming, Yunnan, P.R. China

**Keywords:** FAM136A, lung cancer, immunohistochemistry, proliferation, migration

## Abstract

Lung cancer patients with lymph node metastasis usually had short overall survival and occurred distant metastases at the early stage. However, some of these people did have more prolonged survival. The underlying reason is still unclear. In this study, we found a novel molecule, family with sequence similarity 136, member A gene (FAM136A). First, we performed immunohistochemistry for FAM136A in 177 lung carcinoma tissues. Second, we carried out *in vitro* studies by using A549 and PC-9. We detected FAM136A immunoreactivity in 79 out of 177 (44.6%) lung carcinoma tissues, and the FAM136A status was significantly associated with tumor T stage, lymph node metastasis, and the Tumor-Node-Metastasis (TNM) staging system in these cases. Importantly, it was significantly associated with the overall survival of the patients with lymph node metastasis, especially FAM136A positive patients, who had worse outcomes. Subsequent *in vitro* experiments revealed that the proliferation activity and migration property decreased both A549 and PC-9 lung carcinoma cells transfected with siRNA-FAM136A, and apoptosis reduced. Meanwhile, the expression of CDK4 and CDK6 decreased. FAM136A status would be a potent, worse prognostic factor in lung cancer patients with lymph node metastasis. It would play a vital role in the proliferation, apoptosis, and migration properties of A549 and PC-9. In the future, We will focus on the uncovered signal mechanism between FAM136A and lung cancer.

## Graphical Abstract

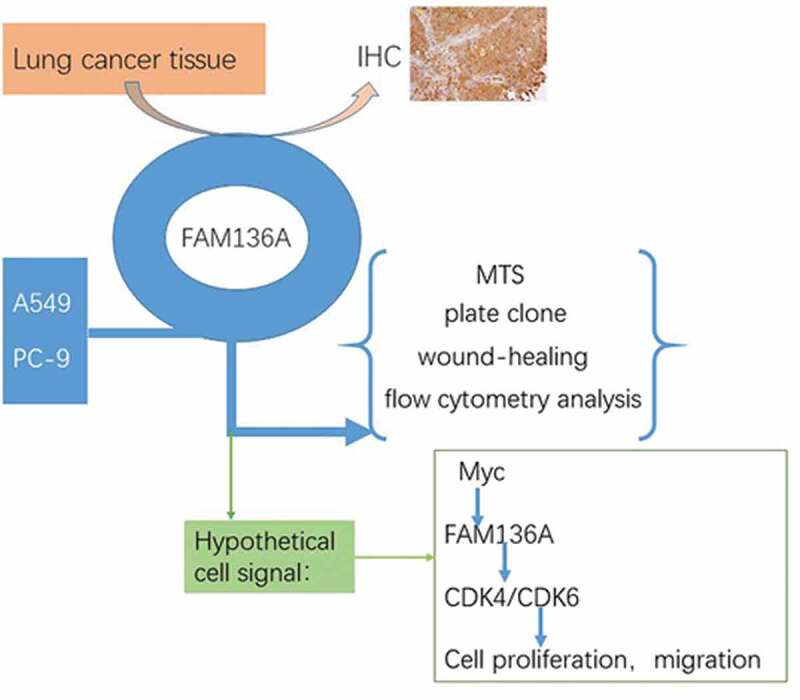

**Abbreviation:** FAM136A: family with sequence similarity 136 member A; NSCLC: non-small-cell lung cancer; SCC: squamous cell carcinoma; IHC: immunohistochemistry; PBS: phosphate-buffered saline; CDK: cyclin-dependent kinase.

## Introduction

Lung cancer is the first most common cancer in the world [1]. The morbidity and mortality of lung cancer are the highest, not only in China but also in the world for both males and females [2]. Although some people who had lymph node metastasis in non-small-cell lung cancer (NSCLC) usually had shorter overall survival, other people had longer overall survival. The reason for this difference is still unknown. Therefore, it is essential to examine novel biological markers in lung cancer to assist us in predicting their prognosis after surgery. In our study, we found a new molecule, which plays a vital role in lung cancer, FAM136A. It is lowly expressed in joint tissues but is highly expressed in various malignant tumor cells [3]. Wang SJ. et al. had previously found that mutations of it in an oral tongue squamous cell carcinoma cell line [4]. It was one of four susceptibility genes(PPP6R2, CANX, FAM136A,and FAM69A) (*p* < 0.05) in ophthalmoplegic subphenotypes of myasthenia gravis (OP-MG), showed altered expression [5]. Also, it was associated with familial Ménière’s disease (MD) [6].

However, FAM136A immunolocalization in lung carcinoma tissue had not been reported until now, its clinical significance remains unclear yet, including overall survival of the patients. Moreover, the biological functions of FAM136A have not been clarified in lung carcinoma cells. Therefore, in this study, we examined FAM136A in lung carcinoma by immunohistochemistry and in vitro studies to explore its clinical and biological significance. Finally, we found that FAM136A activity markedly increased in many lung cancer tissues and cells. FAM136A is considered to play an essential role in lung carcinoma with lymph node metastasis.

Besides, FAM136A is a nuclear-encoded mitochondrial gene downstream targets of Myc [7], which is a transcription factor that binds thousands of genomic Loci in cellular growth control and lymphomagenesis. Myc drove the differential expression of distinct subsets of target genes [8]; we assumed that Myc could drive FAM136A. Myc is a positive regulator of G1-specific cyclin-dependent kinases (CDKs) [8]. Meanwhile, previous studies have shown that activation of cyclin D-Cdk4/6 complexes is current events in a variety of human tumors [9,10]. Also, we have known that Cyclin D/Cdk4 and Cdk6 may conceivably be activated by Myc [11]. We speculated that C-Myc must affect cell cycle progression at additional points downstream of Cdk4 and −6 activation [12].

Interestingly, in this study, we found that the expression level of Cdk4 and −6 was inhibited by using si-FAM136A. It can be speculated that FAM136A could affect Cdk4/6. Therefore, we assume that Myc- FAM136A- CDK4/6 might be a new signaling pathway regulation axis in lung cancer, but this hypothesis needs more evidence.

Finally, this study aimed to find a novel molecule which can distinguish or predict the survival of lung cancer patients with lymph node metastasis, and try to find out the possible regulatory mechanism.

## Materials and methods

### Patients and tissues

One hundred seventy-seven lung carcinoma specimens were acquired from patients who underwent surgical treatment between 2000 and 2017 in the Thoracic Surgery Department of Surgery at Yunnan Tumor Hospital, China (range of age; 27–78). No patients who received chemotherapy or irradiation before surgery were included. The clinical outcome of the patients was evaluated by overall survival, and the mean follow up time was 56 months (range; 1–114 months). All the specimens had been fixed in 10% formalin and embedded in paraffin wax. Research protocols for the present study were approved by the Ethics Committee at Yunnan Tumor Hospital.

### Immunohistochemistry

Samples were processed for immunohistochemical analysis to determine FAM136A expression levels and distribution patterns. Rabbit polyclonal antibodies (ABIN4310204, Sigma, USA) were used to detect FAM136A protein at a 1:50 dilution in phosphate-buffered saline (PBS). The antigen-antibody complex was visualized with 3,3ʹ-diaminobenzidine (DAB, Dako Corporation, Carpinteria, CA, USA) solution and counterstained with hematoxylin. PBS was used as a negative control. Immunoreactivity for FAM136A was detected in the cytoplasm of carcinoma cells, and the sections of the case were scored semi-quantitatively for the extent of immunoreaction as follows: 0, 0% immunoreactive cells; 1, <5% immunoreactive cells; 2, 5–50% immunoreactive cells; and 3, >50% immunoreactive cells. Besides, the intensity of staining was scored semi-quantitatively as 0, negative; 1, weak; 2, intermediate; and 3, strong. The final immunoreaction score was defined as the sum of both parameters (extent and intensity), and samples were grouped according to the summed score as negative (0), weak staining (1–2), moderate staining (3) and intense staining. Final immunoreaction scores >0 were defined as positive. All slides were evaluated independently for protein expression by three separate observers, and slides with incongruent grading were scrutinized a second time, and a consensus was reached.

### Cell lines

Lung cancer cell lines used in this study were A549 and PC-9 (lung adenocarcinoma cell lines) and were provided from the American Type Culture Collection (Manassas, VA, USA). A549 and PC-9 cell lines were grown in DMEM/F12(Invitrogen, Carlsbad, CA, USA), and RPMI 1640(Invitrogen) supplemented with 10% FBS (Sigma-Aldrich, St. Louis, MO, USA)respectively.

### Small interfering RNA (siRNA) transfection

siRNA oligonucleotides for FAM136A were purchased from RiboBio (Guangzhou RiboBio Co., Ltd.). The target sequences of siRNA against FAM136A (si FAM136A) were as follows: 5ʹ- GAAGGAGGCTCTCTTATCA (TT)-3ʹ (sense) and 5ʹ- GGTGCACCATGCATTGCAA (TT)-3ʹ (anti-sense). Stealth RNAi Negative Control (Invitrogen) was also used as a negative control (siCTRL or NC). Cells were transfected with siRNA (final concentration of 10 nM) using Lipofectamine^TM^2000 RNAi transfection reagent (Invitrogen) according to the manufacturer’s protocol.

### Western blot

Western blot was performed as follows with rabbit polyclonal anti-FAM136A antibody (1:500; ABIN4310204, USA), the whole-cell proteins from A549 and PC-9 cells were extracted using Radio-immunoprecipitation assay (RIPA) buffer (Thermo Fisher Scientific Pierce Biotechnology, Rockford, IL, USA). The lysate proteins (50 μg) were subjected to Mini-PROTEAN TGX Precast Gels (Bio-Rad Laboratories, Hercules, CA, USA). Following SDS–PAGE, the proteins were transferred onto Sequi-Blot PVDF (polyvinylidene difluoride) membrane (Bio-Rad). The primary antibody used was anti FAM136A antibody the same as immunohistochemistry (ABIN4310204; Cell Signaling Technology). CDK4 and CDK6 (dilution 1:500) (Abcam, UK). Also, anti-GAPDH (14 C10; Cell Signaling Technology), β-actin (Abcam, UK). The antibody was used as an internal control. (Bio-Rad), and the protein bands were visualized using Image J software (NIH, USA). An HRP-conjugated anti-rabbit IgG antibody was used as the secondary antibody (Zhongshan, Beijing, China). Signals were detected using enhanced chemiluminescence reagents (Pierce, Rockford, IL).

### Cell proliferation assay

A549 and PC-9 cells were transfected with siFAM136A or siCTRL in a 96-well culture plate. The cell proliferation status was quantified by the MTS (3-(4, 5-dimethylthiazol −2-yl)-5-(3-carboxymethoxyphenyl)-2-(4-sulfophenyl)-2 H-tetrazolium, inner salt) method. Briefly, four × 10^3^ cells were plated per well in 96-well culture plates in 150 μl of the medium, and six parallel wells were assigned to each group, as well as a negative control (without cells). Over five days, every 24 h, 30 μl of MTS substrate was added to each well and then incubated for two hours in the darkness. The absorbance at 490 nm was measured for each sample using a plate reader (BMG Labtech, Offenburg, Germany). The concentrations required to inhibit growth by 50% (IC50) were calculated from survival curves using the Bliss method. All experiments were performed three times independently.

### Colony formation assay

Cells at the exponential growth phase were collected from a monolayer culture through trypsinization. Approximately 200 cells were added into each well of a 6-well culture plate in triplicate and cultured in complete culture medium for 14 days. At the end of the experiment, cell colonies were washed twice with PBS, and then fixed in 100% methanol for 15–30 min and stained with Giemsa solution. The number of colonies (≥50 cells) was counted under a microscope, and colony formation was calculated relative to the number of untreated controls. The colony formation efficiency was calculated as follows: Efficiency = (number of colonies/number of cells inoculated) x 100%. Each experiment was repeated once.

### Flow cytometry analysis of cell apoptosis

Cell apoptosis was determined using Annexin V-PE/7ADD Apoptosis Detection Kit (Beijing solarbio science﹠technology co.) according to the manufacturer’s instructions. Briefly, the harvested cells were washed three times in PBS and resuspended in 100 μl binding buffer. The cells were treated with five μl FITC-conjugated Annexin V and ten μl 7-aminoactinomycin-D (7-AAD) Staining Solution and Incubated for 15 min at room temperature, and then resuspended in 100 μl binding buffer. They were finally analyzed using a BD LSR II flow cytometer (BD Biosciences, San Jose, CA).

### Wound-healing assay

For Wound-healing assay, five×10^4^ cells were seeded into each well of culture insert (Corning, USA), and incubated for ten h at 37°C in a 5% CO_2_ atmosphere After cell adherence, Culture Inserts were removed, and the remaining gaps were evaluated under light microscopy and quantified. The relative migration area was then calculated as the ratio of that in the control cells transfected with siCTRL. All assays were independently repeated at least three times.

### Statistical analysis

FAM136A status and clinicopathological factors were evaluated by Student’s t-test or a cross-table using the chi-squared test. Overall survival curves were generated according to the Kaplan-Meier method, and statistical significance was calculated using the log-rank test. Uni- and multivariate analyses were evaluated by a proportional hazard model (Cox). P values<0.05 were considered significant in this study. In the *in vitro* experiments, the statistical analyses were performed using Student’s t-test. SPSS16.0(SPSS Inc., Chicago, IL, USA) and Graph Pad Prism 5.0(GraphPad Software Inc., San Diego, CA, USA) software were used for statistical analyses.

## Results

### Expression and immunolocalization of FAM136A in human lung carcinoma

**Figure 1.**
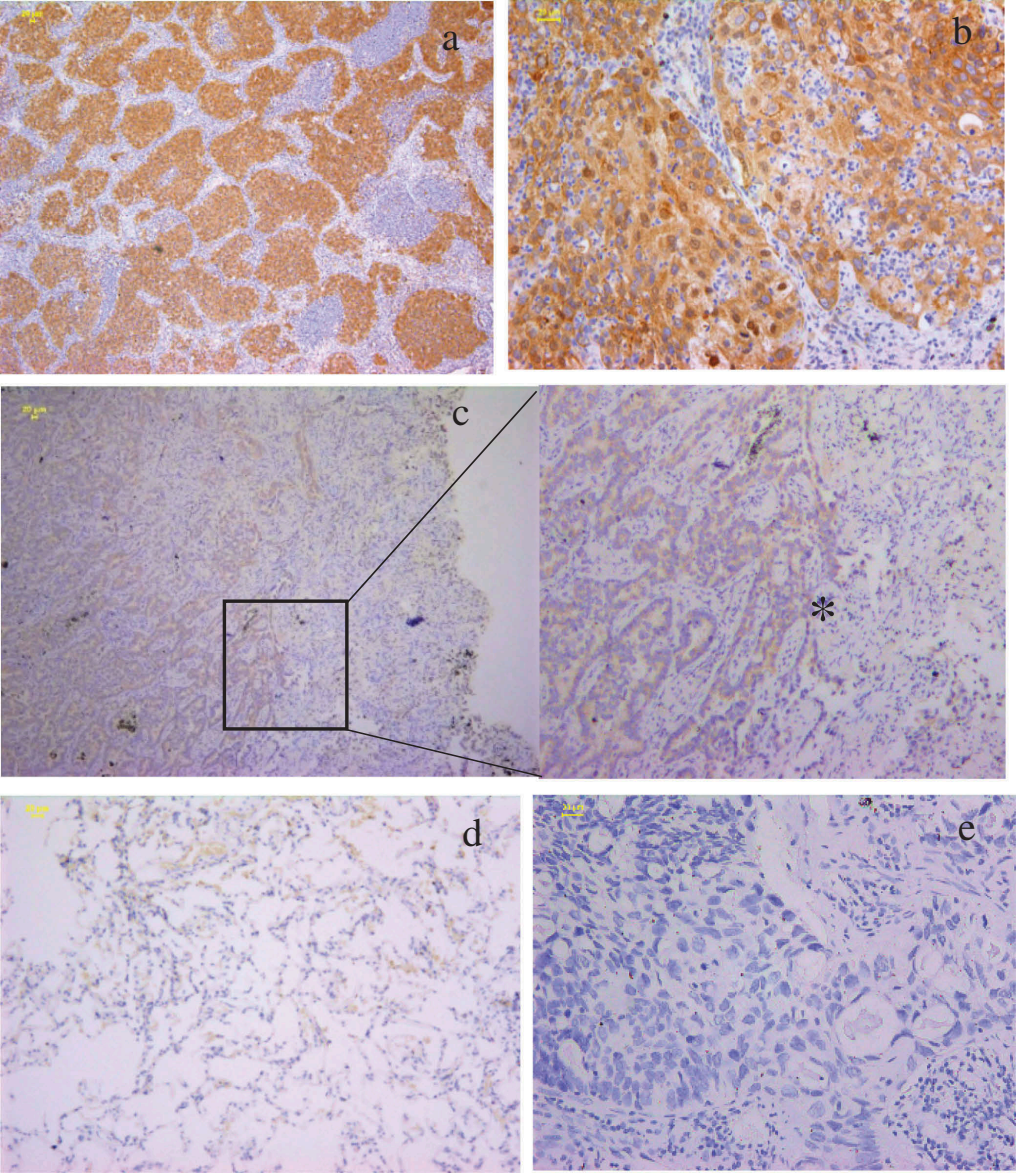
Immunolocalization of FAM136A in human lung carcinoma. **a**. strong immunoreactivity of FAM136A was widely detected in lung carcinoma **b**. FAM136A was immunolocalized mainly in the cytoplasm of lung carcinoma cells. **c**. FAM136A was positive in the in situ lesions of lung carcinoma, but not in the non-neoplastic mucosal epithelium adjacent to the carcinoma (*).**d**. FAM136A was weakly detected in non-neoplastic mucosal epithelium. **e**.Negative control for FAM136A immunohistochemistry. Scale bar: 20 μm.

FAM136A immunoreactivity was widely detected in the cytoplasm of lung carcinoma cells (Figure 1(a,b)) in this study. FAM136A was focally and weakly immunolocalized in the non-neoplastic epithelial cells adjacent to the carcinoma and healthy lung tissue (Figure 1(c,d)). Still, no significant immunoreactivity was detected in the negative control section (Figure 1(e)). Associations between immunohistochemical FAM136A status and various clinicopathological parameters in the lung carcinoma cases were summarized in Table 1. The number of FAM136A -positive cases was 79 out of 177 (44.6%) in this study. The FAM136A status was significantly associated with tumor T stage (P = 0.035), lymph node metastasis (P = 0.046), and the Tumor-Node-Metastasis (TNM) staging system classification [13](P = 0.001). On the other hand, no significant association was detected between FAM136A status and other factors examined, such as patients’ age, gender, and tumor types.

**Table 1.** Association between immunohistochemical FAM136A status and clinicopathological parameters in 177 lung carcinomas.

Clinical pathological parameters and the status of cytoplasmic FAM136A expression by immunohistochemistry				
		FAM136A, *n* (%)		
Variables	Total, *n*	Positive	Negative	*p*-Value
**Age(years)**				
<60	102	44 (42.16)	58 (57.84)	0.54
≥60	75	35 (46.75)	40 (53.25)	
**Gender**				
Male	107	50 (46.30)	57 (53.70)	0.76
Female	70	29 (40.85)	41 (59.15)	
**History of smoking**				
Yes	74	35 (46.05)	41 (53.95)	0.674
No	103	44 (42.72)	57 (57.28)	
**Pathology**				
Adenocarcinoma	133	52 (40.0)	81 (60.0)	0.051
SCC	44	27 (56.82)	17 (43.18)	
**Tumor size**				
T1	102	48 (61.7)	54 (54.2)	0.035*
T2/3/4	75	31 (38.3)	44 (45.8)	
**Lymph node metastasis**				
N0	100	35 (42.0)	65 (58.0)	0.046*
N1/N2	77	44 (57.1)	33 (42.9)	
**AJCC stage**				
Ⅰ	88	28 (35.4)	60 (61.2)	0.001*
Ⅱ-Ⅲ	89	51 (64.6)	38 (38.8)	

*: Data are presented as mean ± SD. All other values represent the number of cases. Statistical analysis was evaluated by the Student’s t-test or a cross-table using the chi-square test. P value<0.05 was considered significant and borderline significant, and are listed in bold. SCC: squamous cell carcinoma.

### Association between FAM136A status and clinical outcome of the patients with lymph node metastasis cases

**Figure 2.**
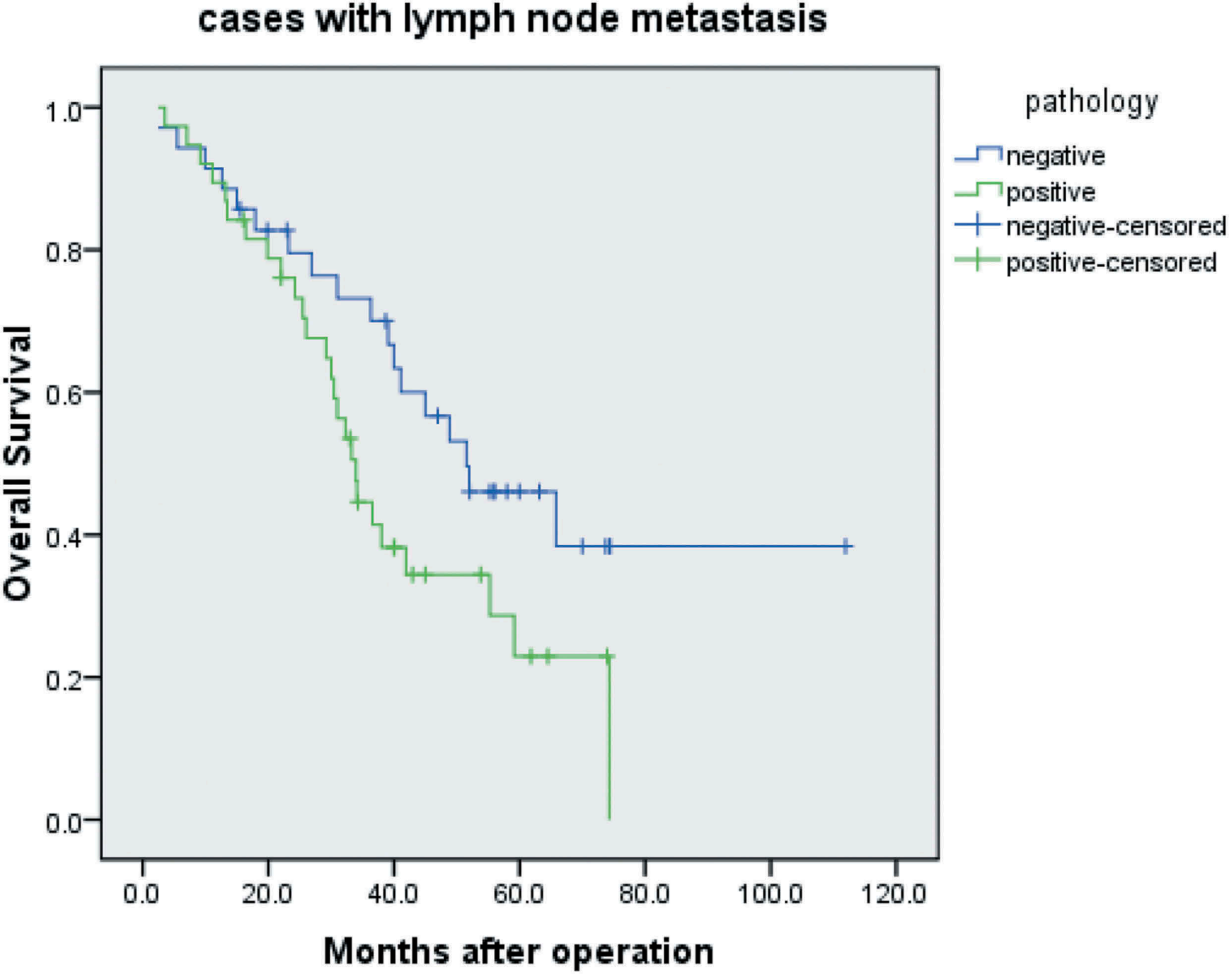
Overall survival of lung cancer patients according to FAM136A status by the Kaplan-Meier method. Stage I–III cases treated by surgery and positive with lymph node metastasis (n = 77). censored:loss to follow-up, Statistical analysis was performed using the log-rank test. P < 0.05.

As shown in Figure 2, FAM136A status was significantly associated with *patients* with lymph node metastasis (P = 0.0085 in overall survival (n = 177). A significant association between FAM136A status and worse prognosis was detected in the lymph node metastasis cases.

The results of the univariate analysis of overall survival using Cox (Table 2), FAM136A status were demonstrated to be significant prognostic factors for overall survival. Multivariate analysis revealed that lymph node metastasis (P = 0.0047) was independent prognostic factors.

**Table 2.** Univariate and multivariate analyses of overall survival in Stage 0 – III lung cancer patients cured by surgery (n = 177).

Variables	Univariate *p-* Value	Multivariate	
		*p*-Value	Relative risk (95%CI)
**FAM136A status (**Positive vs. Negative)	0.001		
**Age (years)** ≥60 vs. <60	0.200		
**Gender (**Male vs. Female)	0.242		
**History of smoking (**yes vs. no)	0.433		
**Tumor size (**T1 vs. T2/3/4)	0.063		
**Lymph node metastasis (**N0 vs. N1/)	0.001	0.004 *	2.530 (1.583–4.043)
**TNM stage (**Ⅰ vs. Ⅱ-Ⅲ)	0.143		

Statistical analysis was evaluated by a proportional hazard model (Cox). Data considered significant (P < 0.05) in the univariate analyses were described as boldface, and these were examined in the multivariate analyses. *: The parameter was categorized into two groups according to the median value. CI: confidence interval.

### Knockdown of FAM136A expression inhibits NSCLC cell proliferation and migration

**Figure 3.**
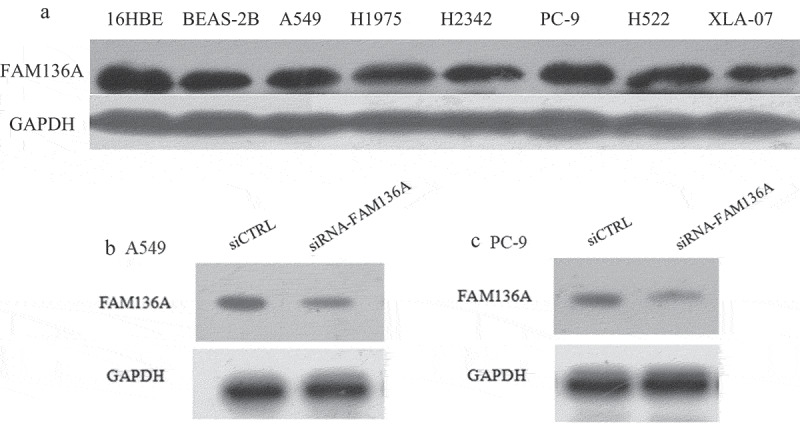
Expression and knockdown of FAM136A expression in NSCLC cell lines. (a) The expression of FAM136A in 7 NSCLC cell lines was detected by Western blotting. (b) and (c).Western blotting analysis of FAM136A expression in A549 and PC-9 cells transfected with FAM136A siRNA. FAM136A siRNA markedly reduced the FAM136A protein level in A549 and PC-9 cells. GAPDH was used as an internal control. 16HBE: Human bronchial epithelial, BEAS-2B: immortalized human bronchial epithelial cells, A549, H1975, H2342, PC-9, H522, XLA-07: human lung adenocarcinoma cell.

The expression of FAM136A was assessed in 6 NSCLC cell lines (i.e., A549, H1975, H2342, PC-9, H522, and XLA-07) (16HBE, BEAS-2B as control)and the FAM136A protein was found to be expressed in all those cell lines (Figure 3(a)). The expression of FAM136A in A549(adenocarcinoma) and PC-9(adenocarcinoma) is at a high level, so A549 and PC-9 cell lines were then selected to assess knockdown of FAM136A expression and the following experiments. The cells were infected with FAM136A-shRNAs/siRNA or negative control shRNA/siRNA(siCTRL). Western blotting data revealed that FAM136A-shRNA significantly reduced the levels of the FAM136A protein compared with siCTRL-transfected A549 cells (Figure 3(b)) and PC-9 cells (Figure 3(c)). (P < 0.01).To further evaluate the biological functions of FAM136A in human lung carcinoma cells, we transfected specific siRNA for FAM136A in A549 and PC-9 lung carcinoma cells in the later experiments.

### The effects of FAM136A expression on cell proliferation in lung carcinoma cells were determined

**Figure 4.**
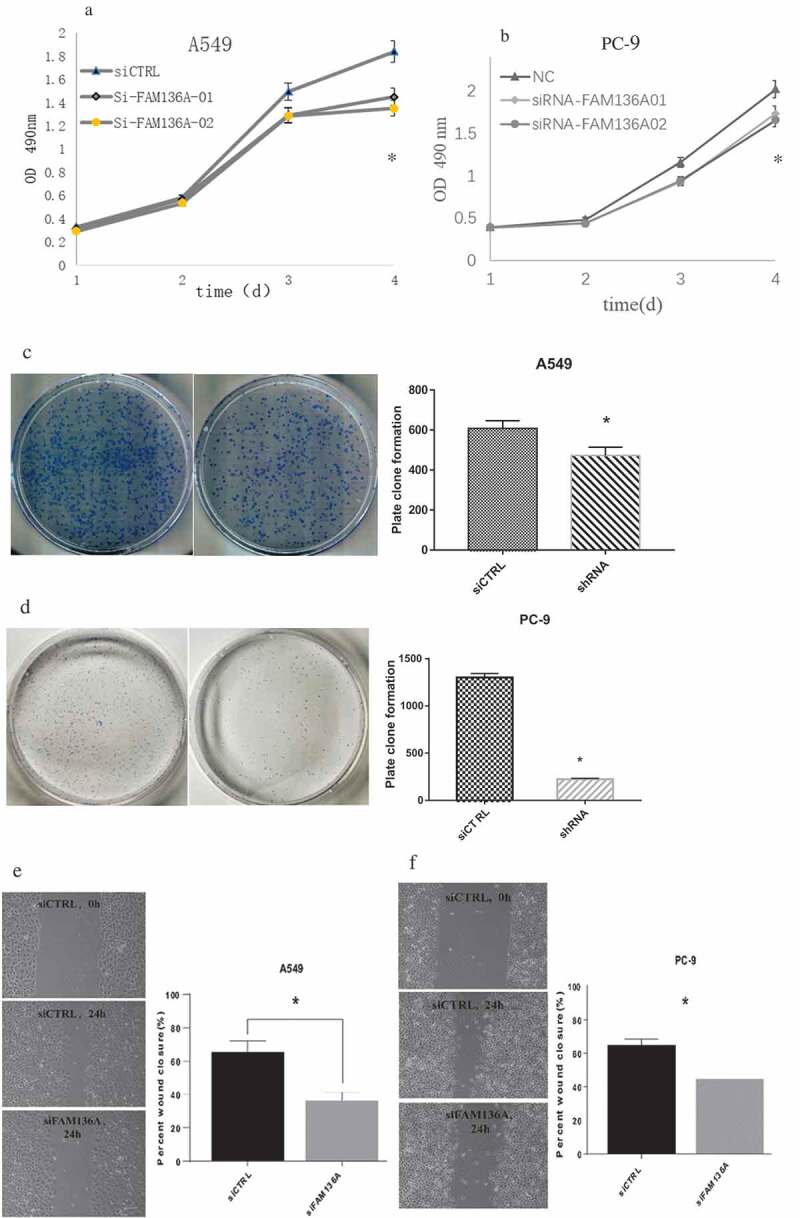
**Knockdown of FAM136A inhibits cell growth and viability of NSCLC cells**. FAM136A knockdown significantly inhibited cell growth in A549 and PC-9 cells detected by MTS assay (Fig a, b). FAM136A knockdown A549 and PC-9 cells formed significantly fewer colonies than those of their control cells (Fig c, d). *p < 0.05 by Student’s t-test. FAM136A. Cell migration was detected by wound-healing assays(Fig. e, f). Bar = 100 μm. In all figures, data were presented as the mean ± SD (n = 3), and the statistical analyses were performed using Student’s t-test. *; P < 0.05, **; P < 0.01 and ***; P < 0.001.

As shown in Figure 4(a), cell proliferation activity was significantly suppressed in A549 cells transfected with siFAM136A from 2 to 4 days after the transfection compared with those transfected with siCTRL (P < 0.001 to P < 0.01 and 0.47- to 0.75-fold from Day 2 to Day 4). Similarly, cell proliferation activity was significantly suppressed in PC-9 cells (P < 0.001 to P < 0.01 and 0.61- to 0.83-fold from Day 2 to Day4 (Figure 4(b)). Moreover, To examine the effect of FAM136A on cell migration, a wound heal assay was used. Relative migration areas in A549 and PC-9 cells transfected with siFAM136A were significantly decreased compared with their controls (A549; P < 0.05, 0.75-fold after 24 hours (Figure 4(e)), PC-9; P < 0.001,0.56-fold) (Figure 4(f)). Furthermore, A549 and PC-9 cells with FAM136A knockdown also formed significantly fewer colonies compared with their control cells (Figure 4(c,d)).

### Knockdown of FAM136A expression induces A549 and PC-9 cell apoptosis

**Figure 5.**
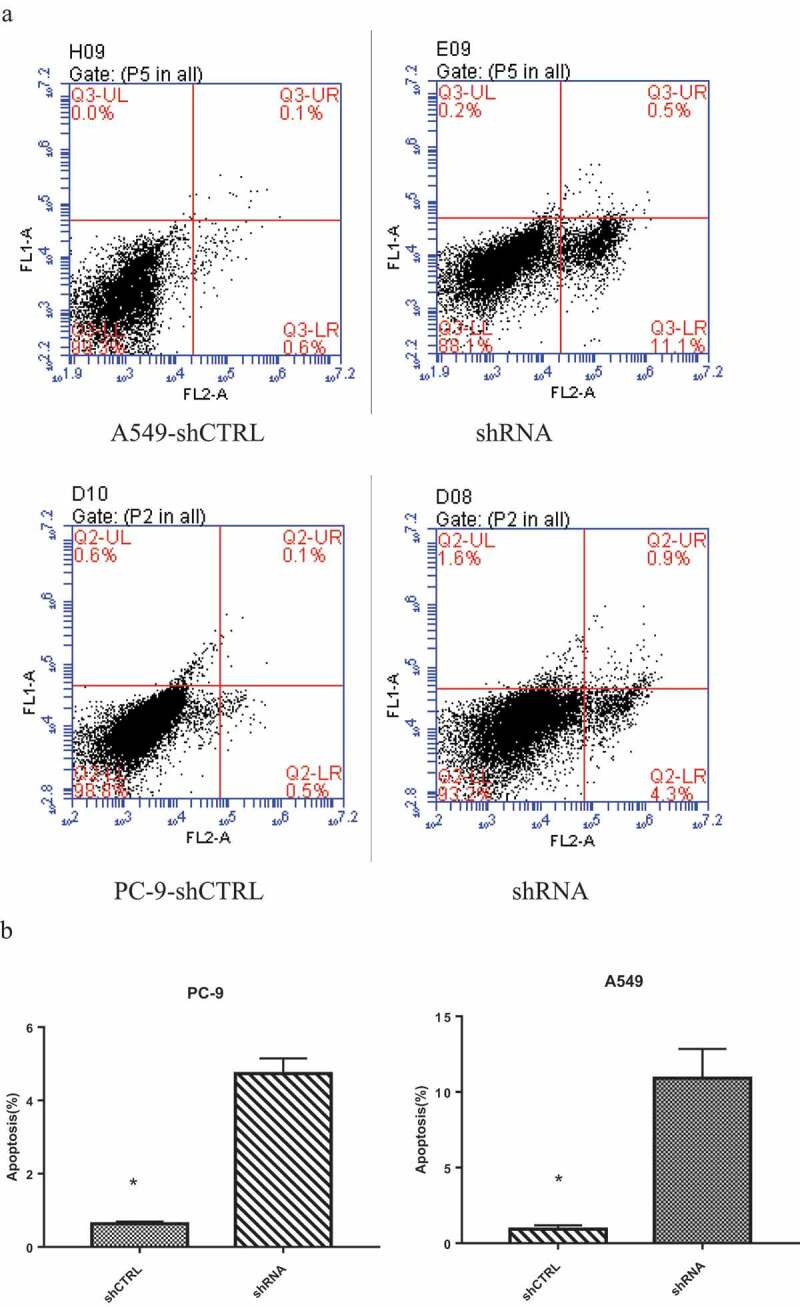
**Knockdown of** FAM136A **expression induced cell apoptosis in NSCLC cells**. The cell apoptosis investigated in A549 and PC-9 cells with FAM136A knockdown and was observed that the apoptosis population was markedly increased compared with their controls. Flow cytometric assay for A549 and PC-9 cells (Fig.a, b). *p < 0.05 by Student’s t-test.

To analyze the effect of cell apoptosis when FAM136A was silenced, a flow cytometric assay was conducted after knockdown of FAM136A expression. As shown in Figure 5, the apoptosis percentage of A549 cells and PC-9 were 11.6% and 5.2% respectively, (0.7% and 0.6%, A549 and PC-9 transfected with siCTRL). All in all, these ﬁndings indicate the shRNA downregulation of FAM136A resulted in cell apoptosis, which contributed to the inhibition of A549 and PC-9 cell growth. Unfortunately, the effect on apoptosis of pc-9 cells was not as significant as that of A549. It could be that the size of pc-9 cells was too small, which affected the transfection efficiency and cell apoptosis.

### Knockdown of FAM136A expression inhibits NSCLC cell proliferation through the downregulation of CDK4 and CDK6 expression

**Figure 6.**
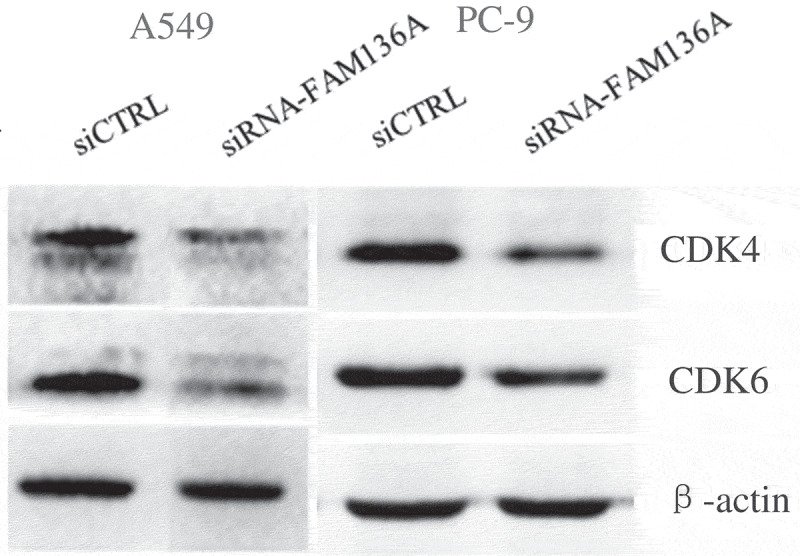
**Expression of cell cycle-related molecules in NSCLC cells with FAM136A knockdown**. CDK4 and CDK6 were significantly downregulated after knockdown of FAM136A expression in A549 and PC-9 cells. *p < 0.05 by Student’s t-test.

To further explore the molecular events underlying the suppression of A549 and PC-9 cell growth following FAM136A knockdown, the expression of CDKs was assessed using western blotting. We found that CDK4 and CDK6 were significantly downregulated after knockdown of FAM136A expression in A549 and PC-9 cells (Figure 6).

## Discussion

Although the FAM136A hypothetical protein-coding gene is conserved across the metazoan, from nematodes to humans [3], we have known little about FAM136A, as mentioned above up till now. FAM136A showed 1.13-fold lower transcriptional levels in chondrocytes from degraded articular cartilage (q = 0.0066) and showed significant evidence of differential expression, which was involved with osteoarthritis [14]. Rare variants in FAM136A and DTNA genes are associated with familial Ménière’s disease (MD) in a Spanish pedigree. It showed an autosomal dominant pattern of inheritance and segregated rare variants with potential pathogenic effects in the genes, including FAM136A [15]. It is a novel gene in the pathways in zebrafish gastrointestinal tract development [16].

Genetic factors are probably relevant in a subset of lung cancer patients with lymph node metastasis [17,18]. The previous study has shown the FAM136A mRNA level was significantly higher in lung carcinoma tissues than the normal lung tissues [19], which is in good agreement with our present findings. In this study, FAM136A immunoreactivity was detected in 44.6% of lung carcinoma cases by using IHC. This is the first study that demonstrates FAM136A as an independent worse prognostic factor of lung carcinoma, which had lymph node metastasis, to the best of our knowledge. Taken together, with these reports and our present results, it is suggested that FAM136A is overexpressed in a subset of lung carcinomas.

Considering that FAM136A immunoreactivity was widely detected in the lung carcinoma in our study, its expression may be upregulated in the process of lung carcinogenesis mainly by various oncogenes, as like Myc. In our present immunohistochemical analysis, FAM136A immunoreactivity was significantly associated with tumor size, lymph node metastasis and TNM stage in Xuanwei lung carcinoma. FAM136A expression was frequently detected in the higher TNM stage in lung carcinoma in our present findings. FAM136A status was significantly associated with worse prognosis in lung cancer patients, and the results of multivariate analyses demonstrated that FAM136A status was an independent prognostic factor for overall survival. Unfortunately, we did not find any correlation between tobacco smoking and FAM136A status.

Moreover, our subsequent *in vitro* experiments demonstrated that both A549 and PC-9 lung carcinoma cells transfected with siRNA against FAM136A significantly decreased cell proliferation and migration properties, but it increased apoptosis. We also found that CDK4/6 expression level reduced by inhibiting FAM136A, but limited information is available about the association between FAM136A and molecular pathways. Myc- FAM136A- CDK4/6 might be a new signaling pathway regulation axis in lung cancer, but this has yet to be demonstrated.

In summary, FAM136A may play a critical role in lung cancer, but further examinations are required to clarify the molecular mechanisms. Since FAM136A possibly involves a variety of biological functions of lung carcinoma cells as described in this section, residual carcinoma cells following surgical treatment in FAM136A- positive lung carcinomas could still have the potential to recur despite the adjuvant therapies rapidly.

Conclusion: FAM136A status is a potent independent prognostic factor in lung cancer patients, especially in those who have lymph node metastasis. CDK4/6 might play a vital role in the proliferation, migration, and apoptosis of lung cancer cells.

## Research Highlights

- FAM136A was highly expressed in Xuanwei lung cancer tissues and was correlated with the T, N stage.
- Knocking down FAM136A inhibited the proliferation and migration of A549 and PC-9.
- Knockdown FAM136A induced cell apoptosis of A549 and PC-9.
- FAM136A might be related to the proliferation, migration, and cell apoptosis of A549 and PC-9 through CDK4 and CDK6.
